# History of the World Allergy Organization: The Miyamoto Years of 1991-1994, ICACI XIV in Kyoto, 1991

**DOI:** 10.1097/WOX.0b013e318221584c

**Published:** 2011-06-15

**Authors:** Terumasa Miyamoto

**Affiliations:** 1 Japan Clinical Allergy Research Institute, Tokyo, Japan

## Abstract

*History of the World Allergy Organization: In 1951, the leaders in allergy from all over the world came together to form the International Association of Allergology and Clinical Immunology (IAACI). For the next 60 years, the allergy world converged at the IAACI triennial meetings, which became biennial in 2003. The international meetings, originally named the International Congress of Allergology and Clinical Immunology (ICACI), are now the World Allergy Congress (WAC) hosted by the World Allergy Organization (WAO). Everyone who has aspired to have worldwide recognition has played a part in IAACI-WAO. The History of the World Allergy Organization traces the global arc of the allergy field over the past 60 years*.

*The current officers of WAO elected to focus on this rich history, inviting prominent leaders who are interested in being part of this history project to write about their time with IAACI-WAO. This series will be presented in Cancún, México as part of the XXII World Allergy Congress (December 4-8, 2011). Leading up to the Congress in Cancún, the World Allergy Organization Journal is presenting segments of the History as part of the "Notes of Allergy Watchers Series," starting with this issue. Please enjoy*.

--*Michael A. Kaliner, MD*

Historian, and Past-President (2006-2007)

World Allergy Organization

## 

At the House of Delegates Meeting of the 12th International Association of Allergology and Clinical Immunology (ICACI) (October 20-25, 1985) in Washington, DC, 5 countries (namely, Australia, Mexico, Sweden, Spain, and Japan) raised their hands to bid to be the host country for ICACI in 1991. Through a vote of the members of the House of Delegates representing the member societies of World Allergy Organization (WAO), Japan was selected to be the host country of ICACI in 1991. From that point forward, members of the Japanese Society of Allergology (JSA) devoted their time preparing to host a successful ICACI XIV (Kyoto 1991).

Kyoto was selected as the congress city for ICACI XIV in 1991 (Figure 1). Members of JSA were appointed to the various committees of the ICACI, such as Organizing, Scientific, Financial, Publication, Social, and Accompanying Ladies Programs, etc., and the Chairs for each committee were also appointed (Figure 2). On July 12, 1989, the 2nd Organizing Committee meeting was held at the Palace Hotel in Tokyo, and Prof. J. Charpin, the officiating President of IAACI at that time, Prof. A. de Weck, Past President of IAACI, and Rick Iber from IAACI Secretariat were invited to join the Japanese Congress Organizing Committee meeting. Our activities for the Congress organization up to that point of time were presented and discussed. The first announcements (40,000 copies) were mailed out in July 1989 and the second announcements (20,000 copies) were mailed out in November 1990. Requests for funds were made to the Japanese Pharmaceutical Manufacturers Association and the Japan Federation of Economic Organization as part of the fund raising activities for the congress toward which we received favorable responses.

**Figure 1:**
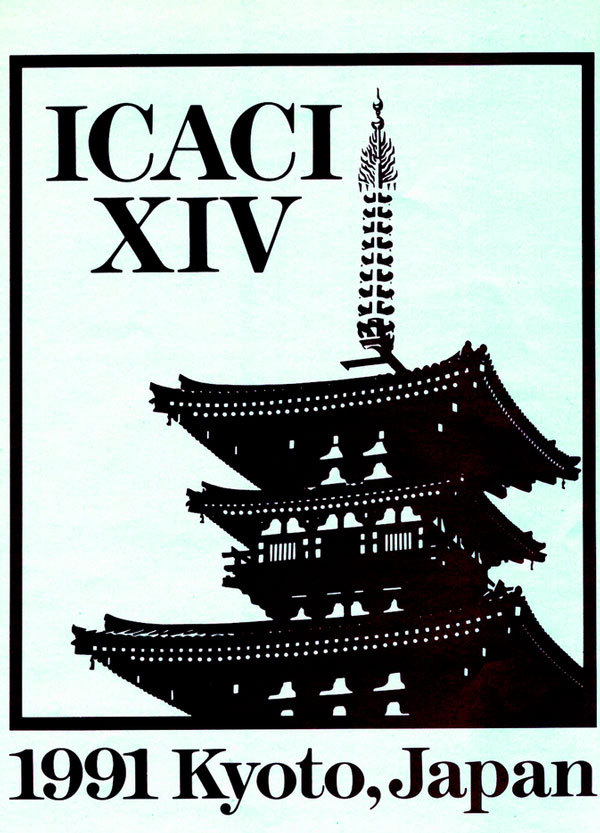
**Cover of program booklet**.

**Figure 2:**
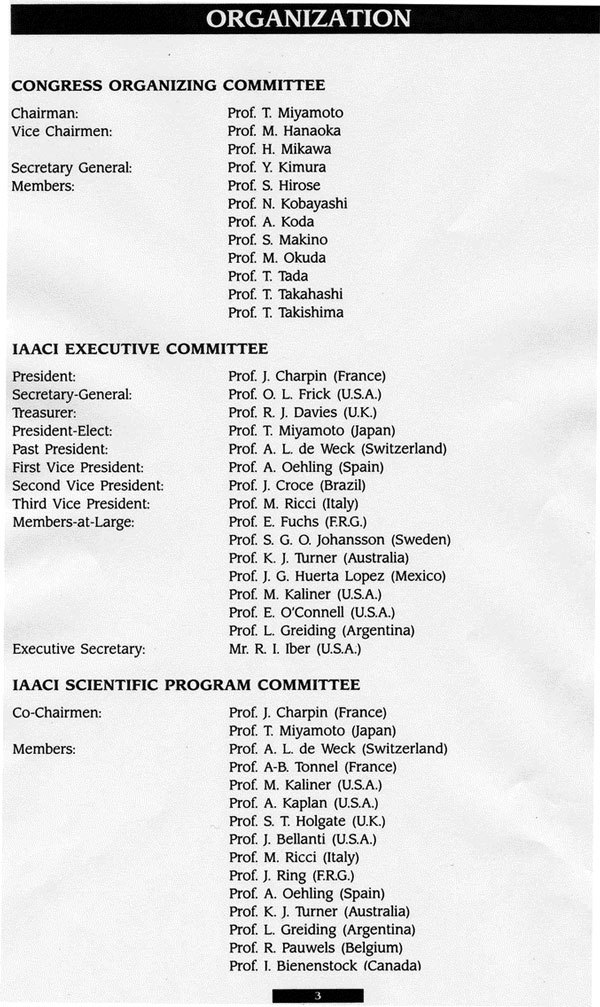
**Names of committee members**.

Members of the Scientific Committee of ICACI XIV were asked to propose titles of symposia and potential candidates as speakers. On July 12, 1990, the Program Committee (namely Prof. T. Tada, Chairman of the Japanese Scientific Committee), myself (from JSA), and invitees (Prof. J Charpin, Prof. A. de Weck, Prof. SGO Johansson, and R. Iber) held a meeting in Munich. At ICACI 1991, we had a total of 1146 abstracts from various countries. The Scientific Committee finalized the titles of the symposia, symposium speakers, and the chairpersons, and the second letters and the invitation letters were sent to the speakers and chairs for their possible acceptance. On April 13 and 14, 1991, the Program Committee held a meeting at Hotel New Otani in Tokyo. Prof. Oehling, Prof. A Kaplan, Prof. R. Goldstein, R. Iber, and the Japanese Scientific Committee members attended the meeting, and the Scientific program for ICACI 1991 was finalized. Young investigators were selected as grant awardees. Because Prof. Charpin resigned from the presidency of IAACI resulting from his physical problems, Prof. A Oehling took over his position to become the new President. The Program was finalized by then, and 5000 copies of the final announcements were mailed out at the end of August 1991. In the meantime, the various local committees met several times to make the Congress quite successful and enjoyable.

The ICACI XIV was held from October 13-18, 1991, in Kyoto at the Kyoto International Conference Centre (KICC) (Figure 3) and the adjacent Prince Hotel. The conference had a total of 3500 delegates from 52 countries (Figure 4). On October 13, the Opening ceremony and Welcome reception were held at the KICCs main congress hall. The Opening Ceremony commenced with a Koto (Japanese harp) performance, followed by the traditional Japanese children's chorus. As President of the Congress, I gave the welcome address and as President of IAACI, Prof. Oehling greeted the delegates and invited guests (Figure 5). A buffet style reception was held to celebrate the start of the congress following to the Opening ceremony. The elaborate cuisine comprised of a combination of Japanese and western style dishes.

**Figure 3:**
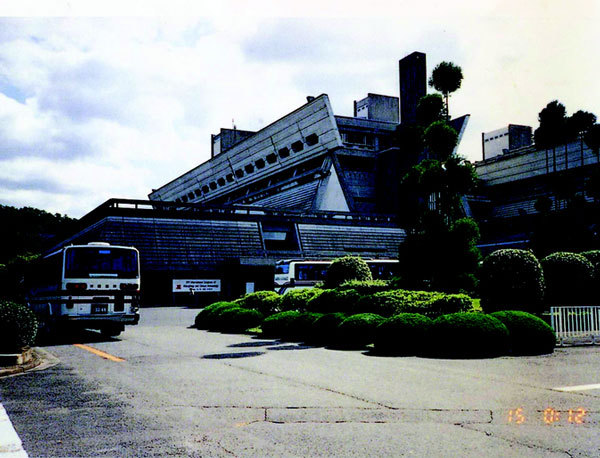
**Kyoto International Conference Center (main hall of Congress)**.

**Figure 4:**
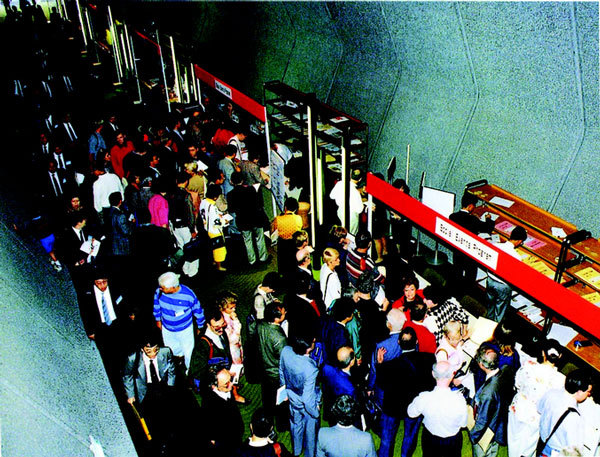
**Reception for registration**.

**Figure 5:**
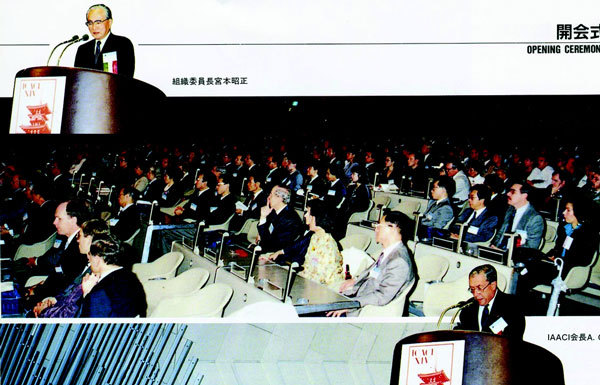
**Opening ceremony**. Above left, T. Miyamoto, Chairman of Organizing Committee. Below right, A. Oehling, President of IAACI.

The Scientific Program comprised of 6 Plenary Lectures, 15 concurrent Symposia, 1 Evening symposium, 46 Meet the Professor sessions, 581 oral presentations, and 583 poster presentations. Forty-five pharmaceutical and manufacturing companies participated in the exhibition to make the Congress more attractive.

The Presidential dinner was held on October 15 at the Kyoto Prince hotel near the congress venue and was attended by 200 invited guests (Figure 6). Several social events were scheduled and many people participated and enjoyed the Japanese culture lecture on Kyoto, the Noh drama, the tea ceremony, flower arrangement demonstrations, pottery, painting, and Japanese sweet making (Figure 7) as well as sightseeing to Kyoto city (Figure 8), west Kyoto garden, east Kyoto garden, Japanese culture course, Nara full day tour, Hozu rapid shooting, and lunch at Shozan. An excursion and get together party was held at Otsu Prince Hotel near Lake Biwa on October 16 with about 1500 participants. The guests were taken to the hotel via 3 routes; namely through Mt. Hiei, Sanju-Sangendo and Kiyomizu temple, and Daigo Sampoin Temple and Fusimi Momoyama castle. From the hotel, the guests enjoyed the excursions by boat on Lake Biwa before the party. The party started with few official addresses, breaking of the Sakadaru (barrel of Japanese Sake) (Figure 9) and Kampai (toast). All delegates and guests spent an enjoyable evening relishing buffet style dishes and drinking various kinds of liquor including sake.

**Figure 6:**
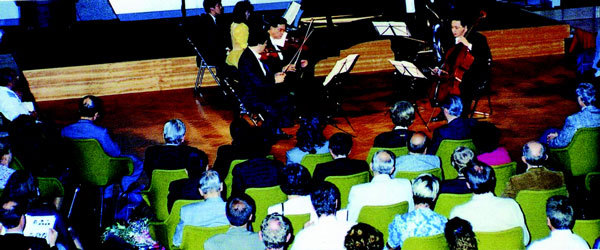
**Concert**.

**Figure 7:**
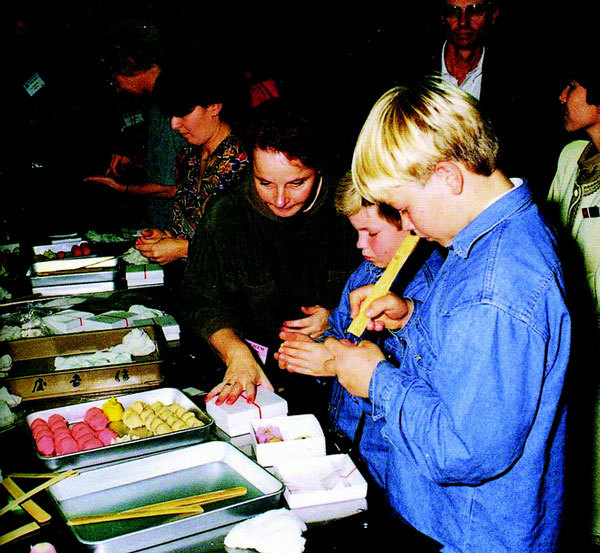
**Japanese sweet making**.

**Figure 8:**
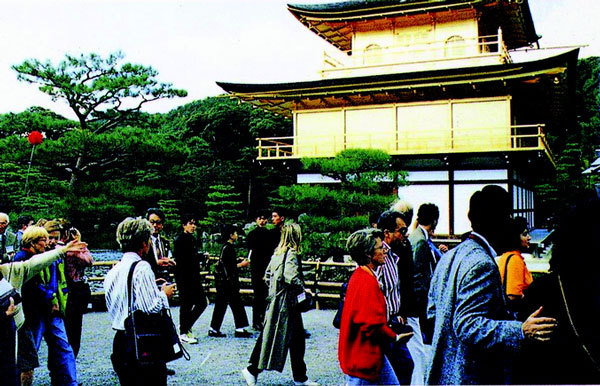
**Sightseeing at Silver Temple in Kyoto**.

**Figure 9:**
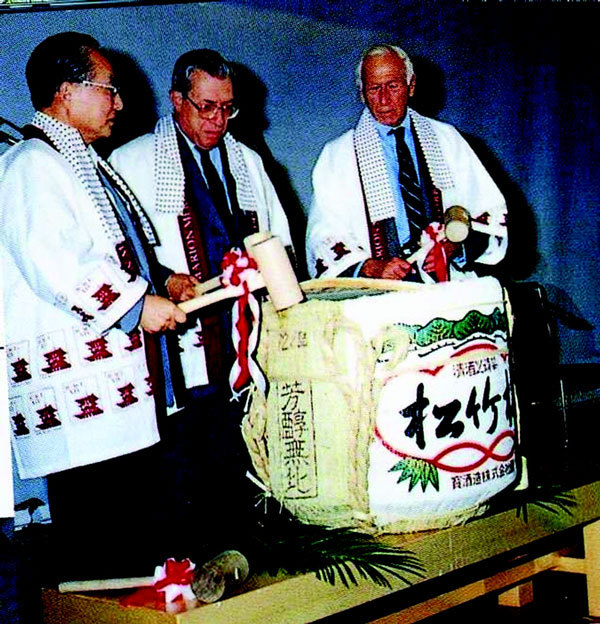
**Breaking of barrel of Japanese sake at get together party**.

A Farewell Awards Reception and Dinner dance was held at the Miyako hotel on October 17 as part of the traditional congress event (Figure 10). Forty five young researchers were awarded for their outstanding contributions to the Congress Scientific Program. Each of them received a waiver of their registration fee and an award amount of Japanese Yen 200,000. Several distinguished scientists and clinicians were awarded the IAACI awards. The Scientific Achievement Award was awarded to Professors T. Kishimoto, L. M. Lichtenstein, A. Barry Kay, and S. Romagnani. The Henry Hyde Salter Clinical Award was awarded to Profs. Jacques Charpin and Albert L. Sheffer. The Distinguished Service Award was presented to Prof. Max Samter.

**Figure 10:**
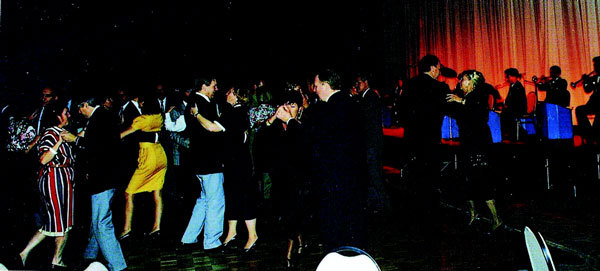
**Dinner dance at farewell**.

During my Presidency, the notable initiative that I and my colleagues at IAACI undertook was the publication of the Membership Directory of IAACI. This project was originally planned by Prof. Charpin, Past President of IAACI. With the collaboration of members of the Executive Committee of IAACI and the help of the member societies, the Membership Directory was published by Hogrefe and Huber in 1992. The publication was supported by a generous contribution from Marion Merrell Dow which enabled the development of the project. In addition, the IAACI Secretariat provided kind assistance in this project. The Directory included a variety of practical items and information that have been very useful and helpful.

